# Early-Phase and Cross-Education Adaptations Following Very Short-Term Unilateral Isokinetic Forearm Extension and Flexion Training in Untrained Women

**DOI:** 10.3390/muscles5010008

**Published:** 2026-01-28

**Authors:** Justin S. Pioske, Jocelyn E. Arnett, Dolores G. Ortega, Trevor D. Roberts, Robert W. Smith, Tyler J. Neltner, Richard J. Schmidt, Terry J. Housh

**Affiliations:** 1 Department of Nutrition and Health Science, University of Nebraska-Lincoln, Lincoln, NE 68520, USA; dortega6@huskers.unl.edu (D.G.O.); troberts17@huskers.unl.edu (T.D.R.); rschmidt1@unl.edu (R.J.S.); thoush1@unl.edu (T.J.H.); 2 Department of Health and Human Performance, Doane University, Crete, NE 68333, USA; jocelyn.arnett@doane.edu; 3 College of Science, Technology, and Health, Lindenwood University, St. Charles, MO 63301, USA; rsmith2@lindenwood.edu; 4 Department of Health and Human Performance, University of Wisconsin-Plattevile, Plattevile, WI 53818, USA; neltnert@uwplatt.edu

**Keywords:** strength, power, rate of velocity development, torque, acceleration

## Abstract

This study: (1) Determined the time course of early-phase adaptations in average peak torque (APT), the rate of velocity development (RVD), and average power (AP) following very short-term unilateral, reciprocal, concentric isokinetic forearm extension and flexion training in untrained women; and (2) determine whether training the non-dominant arm induced cross-education adaptations in the dominant, non-trained arm. Twelve untrained women (age: 21.7 ± 1.2 yrs) completed four testing and four training visits (pre-test and following 2, 3, and 4 days of training). The testing consisted of three maximal repetitions of the dominant and non-dominant arms at 60°, 180°, and 300°·s^−1^, with APT and AP calculated as the average of the 3 repetitions and RVD as the fastest repetition. The training consisted of 6 sets of 10 maximal repetitions at 180°·s^−1^ with the non-dominant arm. The differences in mean values across testing visits for APT, AP, and RVD were determined by separate 2 (Arm) × 2 (Muscle Action) × 3 (Velocity) × 4 (Time [across all testing visits]) repeated measures ANOVA (*α* ≤ 0.05) with Bonferroni-corrected post hoc comparisons. For the trained arm, there were increases in APT (*p* < 0.001) following four training visits and AP following three (*p* = 0.006) and four (*p* = 0.004) training visits. Furthermore, following four training visits, RVD (collapsed across Arms and Muscle Action) decreased at 180°·s^−1^ (*p* = 0.002) and 300°·s^−1^ (*p* = 0.005) following four training visits. There were no changes in APT or AP (*p* = 0.155–1.000) in the non-trained arm, which indicated no cross-education adaptations. These findings suggested that 3–4 days of moderate-velocity, unilateral, reciprocal, isokinetic training elicited early-phase adaptations for APT, RVD, and AP in untrained women, while cross-education adaptations for APT and AP were not observed within this timeframe.

## 1. Introduction

Muscular fitness is characterized by measures of strength, endurance, and power [1], all of which can be improved through an appropriately designed resistance training program. For example, very short-term programs (2 to 3 days) have been used to determine the early-phase muscular adaptations from resistance training and have demonstrated similar increases in muscular strength [2,3,4,5,6,7,8,9] and power [10,11] when compared to longer duration (4 to 10 weeks) training programs [8,12,13,14]. The early-phase adaptations in muscular fitness may have important implications for strength and conditioning coaches as well as allied health professionals such as physical therapists and athletic trainers. For example, enhancing the muscle’s ability to rapidly produce force is a critical objective in athletic development [15,16,17] as well as injury prevention [18,19], rehabilitation [20,21,22], and fall risk assessment among older adults [23]. Furthermore, increases in muscular fitness within a short amount of time may improve adherence to a resistance training or rehabilitation program, and consequently, reduce the risk of reinjury and support continued engagement in exercise and sport activity [18].

Previous very short-term resistance training studies have shown improvements in lower body strength [3,4,5,6,7,8]; however, there is conflicting evidence regarding upper body movements [2,24,25]. The conflicting findings may be attributable to methodological difference, including the training volume (number of training session, sets, repetitions, and load), training velocity (e.g., 60°·s^−1^, 180°·s^−1^, or 300°·s^−1^), sex of the subjects (men versus women), and muscle action performed (non-reciprocal concentric versus reciprocal concentric). For example, Traylor et al. [9,11,25] examined 3 days of training with five sets of 10 non-reciprocal, concentric (agonist concentric muscle action from a static start or following a passive lengthening of the agonist due to gravity), isokinetic forearm flexion (FLEX) repetitions a 60°·s^−1^ and reported increases in peak torque in untrained men, but not untrained women. Furthermore, Cunha et al. [7] compared 3 days of non-reciprocal, concentric, isokinetic leg extension (EXT) training to reciprocal, concentric (rapid transitions from the antagonist concentric muscle action to the agonist concentric muscle action), isokinetic leg EXT and FLEX training and reported the following: (1) the reciprocal group produced greater peak torque at 60°·s^−1^ than the non-reciprocal group, and (2) only the reciprocal group increased peak torque at 180°·s^−1^. Beck et al. [24], however, examined the effects of reciprocal, concentric, isokinetic forearm EXT and FLEX training with six sets of 10 repetitions at 180°·s^−1^ and reported no changes in peak torque in untrained men following 2 days of training. Therefore, the minimum number of reciprocal, concentric, isokinetic forearm FLEX and EXT training sessions required to elicit early-phase muscular adaptations in women remains unknown.

Brown and Whitehurst [26] described changes in limb acceleration, which they termed the rate of velocity development (RVD), as analogous to the rate of force development. The RVD is the time required to reach the load range phase during isokinetic dynamometry, where quantifiable torque can be measured. Brown and Whitehurst [26] hypothesized that previously reported early-phase increases in muscular strength [8] may be attributable to the failure to control for the acceleration phase of the movement, which can produce artificial increases in torque [27]. Therefore, the assessment of RVD may partially explain the early-phase increases in peak torque production. Furthermore, RVD has been recognized as a key indicator of muscle quality [28,29,30] and functional performance [31,32,33,34,35]. For example, Mota et al. [29] and Hirata et al. [28] found muscle quality to be negatively correlated with RVD in elderly men and suggested that qualitative changes in contractile properties in muscle may influence the ability to rapidly accelerate the limb. Furthermore, Wang et al. [30] found that 8 weeks of dynamic constant external resistance (DCER) training with fast velocities (0.5 s concentric) resulted in greater improvements in leg extensor RVD and muscle quality than training with slow velocities (3 s concentric) in untrained women. Murry et al. [33], however, found that 4 weeks of isokinetic training at slow (60°·s^−1^) and fast (400°·s^−1^) velocities improved long jump performance and RVD in recreationally active men. Therefore, the capacity to rapidly contact and accelerate a limb is an important aspect of muscle function across the life span. In addition, previous studies [25,31,32,33,36] have suggested that RVD may be a useful tool for identifying neuromuscular performance changes. To date, however, only Brown and Whitehurst [26] have investigated the early-phase RVD adaptations in the leg extensors and reported reductions following 2 days of isokinetic training. The time course of early-phase RVD adaptations of the forearm flexors and extensors, however, has not been determined.

Power (*P*) is the time derivative of work (*P* = Δwork/Δtime), while work (*W*) is the product of the total force (*F*) and the displacement (Δ*d*) through which the force is applied (*W* = F × Δ*d*) [37]. Therefore, muscular power can be simplified as the product of force and velocity (*V*) vectors (*P* = *F* × [Δ*d*/Δtime] = *F* × *V*) and is equally influenced by changes in either force or velocity [37]. Furthermore, it has been suggested that muscular power is a better criterion than muscular strength for determining when to return to sport participation following an injury [20] as well as preserving functional independence in older adults [38]. Few studies [10,11], however, have examined the early-phase adaptations in muscular power. For example, Cramer et al. [10] examined the effects of 3 days of concentric, isokinetic leg EXT training with 3 sets of 10 repetitions at 150°·s^−1^ in recreationally active men and found no change in muscular power, despite increases in peak torque and decreases in RVD. Traylor et al. [11], however, investigated the effects of 3 days of concentric, isokinetic forearm FLEX training with 5 sets of 10 repetitions at 60°·s^−1^ and found 8 to 20% increases in muscular power in untrained men, but not in untrained women. Furthermore, Garnica [14] found 29% and 30% increases in muscular power at 60°·s^−1^ and 180°·s^−1^ following 4 weeks of reciprocal, concentric isokinetic shoulder FLEX and EXT training with four sets of five repetitions in untrained women. The early-phase muscular power adaptations following reciprocal, concentric, isokinetic training of the forearm flexors and extensors at a moderate velocity, however, have not yet been investigated in untrained women.

Cross-education [39] is a phenomenon where neurally mediated adaptations that occur in the trained limb transfer to the non-trained limb following unilateral resistance training [40]. It is theorized that unilateral resistance training (1) activates neural circuits that project to both the contralateral and ipsilateral limb, leading to increased muscular function [41,42]; and/or (2) induces adaptations in motor areas of the brain that are primarily involved in the control of movements, thus allowing for the non-trained limb to access these modified neural circuits in ways similar to motor learning [43,44]. Therefore, cross-education may be helpful for maintaining the muscular integrity of an immobilized limb following injury [45,46]. Farthing et al. [47,48] reported that cross-education-induced increases in muscular strength in the non-trained limb occurred only when the dominant limb was trained. More recent studies [49,50], however, have reported cross-education adaptations in muscular strength in the dominant, non-trained limb following ≥3 weeks of unilateral resistance training with the non-dominant limb. There is, however, a need to evaluate the time-course of cross-education adaptations produced by unilateral training [39]. To date, only Costa et al. [5] have examined the early-phase cross-education adaptations following 3 days of unilateral DCER leg extension training with the dominant leg and reported increases in strength for both the trained and non-trained legs. No previous studies, however, have examined the early-phase cross-education adaptations following unilateral forearm isokinetic resistance training with the non-dominant arm. Therefore, the purposes of the present study were twofold: (1) to determine the time course of early-phase adaptations in muscular strength, RVD, and power following 2, 3, and 4 days of unilateral, reciprocal, concentric, isokinetic forearm FLEX and EXT training at 180°·s^−1^ in untrained women; and (2) to determine whether very short-term resistance training with the non-dominant arm results in cross-education adaptations in the dominant, non-trained arm. Based on previous findings [3,5,10,11,24,25,26,39], it was hypothesized that 2, 3, and/or 4 days of training with reciprocal muscle actions at 180°·s^−1^ would elicit increases in muscular strength and power as well as decreases in RVD in the trained and non-trained arm.

## 2. Results

### 2.1. Reliability

The test–retest reliability parameters (*p*-value for mean differences, intraclass correlation coefficient [ICC], ICC 95% confidence interval [ICC_95%_], standard error of measurement [SEM], and minimal difference [MD]) for average peak torque (APT), average power (AP), and RVD of the trained arm and non-trained arm are presented in Table 1 (EXT) and Table 2 (FLEX). For the trained arm, there were no significant mean differences for test versus retest for APT at 60°·s^−1^ and 300°·s^−1^ for both muscle actions (ICC = 0.889 to 0.985), AP at 180°·s^−1^ and 300°·s^−1^ for EXT (ICC = 0.919 to 0.970), AP for FLEX across all velocities (ICC = 0.943 to 0.978), as well as RVD across all velocities and muscle actions (ICC = 0.661 to 0.890). For the non-trained arm, there were no significant mean differences for APT across all velocities and muscle actions (ICC = 0.907 to 0.952), AP at 60°·s^−1^ and 300°·s^−1^ for both muscle actions (ICC = 0.897 to 0.959), AP at 180°·s^−1^ for EXT (ICC = 0.975), RVD at 180°·s^−1^ and 300°·s^−1^ for both muscle actions (ICC = 0.283 to 0.873), and RVD 60°·s^−1^ for EXT (ICC = 0.429). There were, however, significant mean differences for the trained arm for APT at 180°·s^−1^ for FLEX and EXT (*p* = 0.042, ICC = 0.928, and *p* = 0.015, ICC = 0.992, respectively), and AP at 60°·s^−1^ for EXT (*p* = 0.038, ICC = 0.920). In addition, there were significant mean differences for the non-trained arm for AP at 180°·s^−1^ for FLEX (*p* = 0.011, ICC = 0.939), and RVD at 60°·s^−1^ for FLEX (*p* = 0.043, ICC = 0.601).

The SEM for APT and AP of the trained arm ranged from 0.7 to 2.7 Nm and 1.7 to 4.8 W, respectively, across all velocities and muscle actions. The SEM for RVD of the trained arm ranged from 14.3 to 82.0 ms, with greater error observed at faster velocities during FLEX compared to EXT. For the non-trained arm, the SEM for APT and AP across all velocities and muscle actions ranged from 1.6 to 2.8 Nm and 2.0 to 14.4 W, respectively. The SEM for RVD of the non-trained arm ranged from 20.3 to 75.5 ms, with a greater error observed with faster velocities for EXT, but not for FLEX (60°·s^−1^ = 21.6 ms, 180°·s^−1^ = 72.7 ms, and 300°·s^−1^ = 20.3 ms).

The MD to be considered a ‘real’ change [51] for APT and AP of the trained arm ranged from 3.0 to 7.6 Nm and 4.7 to 10.5 W, respectively, across all velocities and muscle actions. The MD for RVD of the trained arm ranged from 39.7 to 67.7 ms across all velocities for EXT, while FLEX ranged from 92.6 to 227.3 ms across all velocities. The MD for the non-trained arm ranged from 4.5 to 7.7 Nm and 5.5 to 12.1 W for APT and AP, respectively, across all velocities and muscle actions. The MD for RVD of the non-trained arm ranged from 56.3 to 180.1 ms for EXT across all velocities, while FLEX ranged from 59.8 to 209.3 ms across all velocities.

### 2.2. Analysis of Variance

#### 2.2.1. Average Peak Torque

The repeated measures ANOVA for APT revealed a significant four-way interaction for Arm × Muscle Action × Velocity × Time (*p* = 0.044 $\eta_{p}^{2}$ = 0.173) that was decomposed by Arm. For the trained arm, the results of the repeated measures ANOVA revealed a significant main effect for Time (*p* = 0.001, $\eta_{p}^{2}$ = 0.375). Post hoc, pairwise comparisons with Bonferroni corrections (*p* ≤ 0.05/6 = 0.008) for the main effect for Time (collapsed across Muscle Action and Velocity) revealed that APT was significantly greater following Day 4 of training (21.3 ± 6.5 Nm) than following Day 2 of training (19.3 ± 5.8 Nm, *p* < 0.001, *d* = 1.360), but not significantly greater than pre-test (18.1 ± 4.8 Nm, *p* = 0.014, *d* = 0.841) or following Day 3 of training (20.4 ± 5.9 Nm, *p* = 0.063, *d* = 0.597). For the non-trained arm, the repeated measures ANOVA revealed no significant three-way interaction, two-way interaction, or main effect for Time (*p* = 0.074 to 0.995, $\eta_{p}^{2}$ = 0.002 to 0.200).

#### 2.2.2. Rate of Velocity Development

The repeated measures ANOVA for RVD revealed significant two-way interactions for Muscle Action × Velocity (*p* < 0.001, $\eta_{p}^{2}$ = 0.777), Muscle Action × Time (*p* = 0.050, $\eta_{p}^{2}$ = 0.208), and Velocity × Time (*p* = 0.016, $\eta_{p}^{2}$ = 0.205). The two-way interaction for Muscle Action × Velocity was decomposed by Muscle Action. For EXT (collapsed across Arm and Time), the repeated measures ANOVA revealed a significant main effect for Velocity (*p* < 0.001, $\eta_{p}^{2}$ = 0.949). Post hoc, Bonferroni-corrected pairwise comparisons (*p* ≤ 0.05/3 = 0.017) revealed that RVD was significantly faster at 60°·s^−1^ (79.0 ± 13.0 ms) than 180°·s^−1^ (158.4 ± 30.7 ms, *p* < 0.001, *d* = 2.879) and 300°·s^−1^ (245.0 ± 39.0 ms, *p* < 0.001, *d* = 5.456). In addition, 180°·s^−1^ (158.4 ± 30.7 ms) was significantly faster than 300°·s^−1^ (245.0 ± 39.0 ms, *p* < 0.001, *d* = 3.227). For FLEX (collapsed across Arm and Time), the repeated measures ANOVA revealed a significant main effect for Velocity (*p* < 0.001, $\eta_{p}^{2}$ = 0.937). Post hoc, Bonferroni-corrected pairwise comparisons (*p* ≤ 0.05/3 = 0.017) revealed that RVD was significantly faster at 60°·s^−1^ (107.5 ± 25.5 ms) than 180°·s^−1^ (235.4 ± 52.4 ms, *p* < 0.001, *d* = 3.121) and 300°·s^−1^ (398.6 ± 89.8 ms, *p* < 0.001, *d* = 3.839). In addition, 180°·s^−1^ (235.4 ± 52.4 ms) was significantly faster than 300°·s^−1^ (398.6 ± 89.8 ms, *p* < 0.001, *d* = 3.750).

The two-way interaction for Muscle Action × Time was decomposed by Muscle Action. For EXT (collapsed across Arm and Velocity), the repeated measures ANOVA revealed a significant main effect for Time (*p* = 0.005, $\eta_{p}^{2}$ = 0.321); however, the post hoc, Bonferroni-corrected pairwise comparisons (*p* ≤ 0.05/6 = 0.008) revealed no significant differences (*p* > 0.008) between pre-test (168.8 ± 23.4 ms), following Day 2 (166.1 ± 30.5 ms), Day 3 (160.1 ± 28.8 ms), or Day 4 (148.2 ± 26.7 ms) of training. For FLEX (collapsed across Arm and Velocity), the repeated measures ANOVA revealed a significant main effect for Time (*p* < 0.001, $\eta_{p}^{2}$ = 0.398). Post hoc, Bonferroni-corrected pairwise comparisons (*p* ≤ 0.05/6 = 0.008) revealed that RVD was significantly faster following Day 4 of training (220.78 ± 51.4 ms) than at pre-test (277.9 ± 71.5 ms, *p* = 0.002, *d* = 1.207) and following Day 2 of training (253.2 ± 60.2 ms, *p* = 0.006, *d* = 0.920), but not significantly different than following Day 3 of training (204.2 ± 52.2 ms, *p* = 0.149, *d* = 0.416).

The two-way interaction for Velocity × Time was decomposed by Velocity. For 60°·s^−1^ (collapsed across Arm and Muscle Action), the repeated measures ANOVA revealed no significant main effect for Time (*p* = 0.304, $\eta_{p}^{2}$ = 0.103). For 180°·s^−1^ (collapsed across Arm and Muscle Action), the repeated measures ANOVA revealed a significant main effect for Time (*p* = 0.002, $\eta_{p}^{2}$ = 0.103). Post hoc, Bonferroni-corrected pairwise comparisons (*p* ≤ 0.05/6 = 0.008) revealed that RVD was significantly faster following Day 4 of training (170.4 ± 34.0 ms) than pre-test (218.5 ± 50.5 ms, *p* = 0.005, *d* = 1.016), but not following Day 2 (199.8 ± 43.0 ms, *p* = 0.015, *d* = 0.828) or Day 3 (199.0 ± 48.7 ms, *p* = 0.009, *d* = 0.912) of training. For 300°·s^−1^ (collapsed across Arm and Muscle Action), the repeated measures ANOVA revealed a significant main effect for Time (*p* < 0.001, $\eta_{p}^{2}$ = 0.392). Post hoc, Bonferroni-corrected pairwise comparisons (*p* ≤ 0.05/6 = 0.008) revealed that RVD was significantly faster following Day 4 of training (296.9 ± 67.4 ms) than pre-test (353.1 ± 74.4 ms, *p* = 0.002, *d* = 1.153) and following Day 2 of training (334.2 ± 71.6 ms, *p* = 0.003, *d* = 1.098), but not following Day 3 of training (303.1 ± 59.5 ms, *p* = 0.714, *d* = 0.109).

#### 2.2.3. Average Power

The repeated measures ANOVA for AP revealed a significant two-way interaction for Arm × Time (*p* = 0.019, $\eta_{p}^{2}$ = 0.257) that was decomposed by Arm. For the trained arm (collapsed across Muscle Action and Velocity), the repeated measures ANOVA revealed a significant main effect for Time (*p* < 0.001, $\eta_{p}^{2}$ = 0.468). Post hoc, pairwise comparisons with Bonferroni corrections (*p* ≤ 0.05/6 = 0.008) revealed that AP following Day 4 of training (24.6 ± 11.3 W) was significantly greater than pre-test (18.4 ± 7.6 W, *p* = 0.004, *d* = 1.052) and following Day 2 (21.7 ± 10.6 W, *p* = 0.003, *d* = 1.082) of training. In addition, the post hoc, pairwise comparisons revealed that AP was significantly greater following Day 3 of training (23.3 ± 10.0 W) than pre-test (18.4 ± 7.6 W, *p* = 0.006, *d* = 0.975), but not following Day 2 of training (21.7 ± 10.6 W, *p* = 0.049, *d* = 0.639). For the non-trained arm (collapsed across Muscle Action and Velocity), the repeated measures ANOVA revealed a significant main effect for Time (*p* = 0.009, $\eta_{p}^{2}$ = 0.291); however, the post hoc, Bonferroni-corrected pairwise comparisons revealed no significant differences (*p* > 0.008) between pre-test, following Day 2, Day 3, or Day 4 of training.

## 3. Discussion

### 3.1. Reliability of Testing Procedures

The test–retest reliability analyses for APT and AP of the trained arm demonstrated excellent reliability (ICC = 0.889 to 0.992) for both muscle actions and across all velocities (Table 1). There were, however, significant mean differences observed for EXT and FLEX APT at 180°·s^−1^, EXT AP at 60°·s^−1^, and FLEX AP at 180°·s^−1^ despite demonstrating excellent reliability (ICC = 0.920 to 0.992). Therefore, the subjects in the present study maintained their rank order relative to others but demonstrated a systematic shift in test–retest scores. It is possible that the subjects became more familiarized with the test, despite having prior familiarization to the testing apparatus. The ICC, however, only assesses the reliability of test scores between subjects, whereas the SEM assesses the reliability of individual test scores, regardless of the sample population [51]. The SEM, therefore, provides an additional index for assessing the reliability of test versus retest scores by quantifying the variability of the observed score from the true score due to measurement error. The SEM (expressed as a percentage of the pre-test value) for EXT and FLEX APT at 180°·s^−1^ were 11.0% and 3.1% of the pre-test value, respectively. The SEM for EXT AP at 60°·s^−1^ and FLEX AP at 180°·s^−1^ was 12.5% and 9.9% of the pre-test value, respectively. For the non-trained arm, the test–retest reliability demonstrated excellent reliability (ICC = 0.907 to 0.975) for APT and AP with SEM ranging from 7.3% to 20.6% of the pre-test value for both muscle actions and all velocities. There were, however, significant mean differences observed for AP at 180°·s^−1^ for FLEX (SEM = 13.7% of the pre-test value) despite demonstrating excellent reliability (ICC = 0.939). Similar to the test–retest results for the trained arm, the systematic shift in test–retest scores for the non-trained arm may be due to the greater familiarity with the testing apparatus, despite the prior familiarization session.

The test–retest reliability analyses for RVD for the trained arm and non-trained arm revealed no significant mean differences between baseline and pre-test values for both muscle actions and across all velocities. The trained arm demonstrated good and excellent reliability for EXT and FLEX at 60°·s^−1^ (ICC = 0.661 and 0.788, respectively) and excellent reliability for both muscle actions at 180°·s^−1^ and 300°·s^−1^ (ICC = 0.765 to 0.890). The SEM for RVD of the trained arm for both muscle actions presented a greater range of error (relative to the pre-test value) at slower velocities (60°·s^−1^: 16.3% to 25.6%) than for faster velocities (180°·s^−1^: 12.9 to 22.8%; and 300°·s^−1^: 8.2% to 16.6%). For the non-trained arm, however, the test–retest reliability analyses for RVD demonstrated fair reliability for EXT and FLEX at 60°·s^−1^ (ICC = 0.429 and 0.601, respectively) and for FLEX at 180°·s^−1^ (ICC = 0.686), poor reliability for EXT at 180°·s^−1^ (ICC = 0.283), and excellent reliability for EXT and FLEX at 300°·s^−1^ (ICC = 0.791 and 0.873, respectively). The SEM for RVD of the non-trained arm across both muscle actions presented larger range of error (relative to the pre-test values) at 60°·s^−1^ (SEM = 22.7% to 50.1%), 180°·s^−1^ (SEM = 30.4% to 46.2%) and 300°·s^−1^ (SEM = 9.0% to 18.9%) compared to the trained arm (60°·s^−1^: 16.3% to 25.6%; 180°·s^−1^: 12.9 to 22.8%; and 300°·s^−1^: 8.2% to 16.6%). The reliability for RVD in the present study were consistent with the findings of previous studies [26,52] that reported lower ICC values for RVD at slower velocities (i.e., 60°·s^−1^; ICC = 0.400) than higher velocities (i.e., 240°·s^−1^; ICC = 0.870), as well as lower percent error at higher velocities (i.e., 240°·s^−1^; SEM = 3.2%) than for slower velocities (i.e., 60°·s^−1^; SEM = 9.6%) from performing maximal, reciprocal, concentric, isokinetic leg EXT and FLEX testing.

### 3.2. Early-Phase Adaptations in the Trained Arm

#### 3.2.1. Average Peak Torque

**Figure 1:**
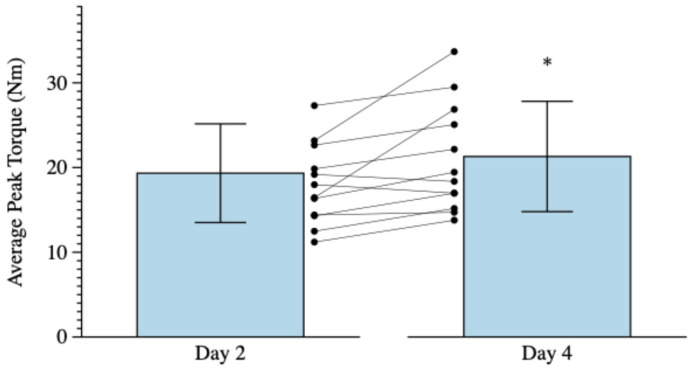
Trained arm marginal means (±SD) and individual spaghetti graphs for average peak torque (collapsed across Velocity and Muscle Action) following Day 2 of training and following Day 4 of training. * Day 4 > Day 2 (*p* < 0.001).

The present study investigated the time course of early-phase adaptations in APT, RVD, and AP following 2, 3, and 4 days of unilateral, reciprocal, concentric, isokinetic forearm EXT and FLEX training at 180°·s^−1^ in untrained women. There were training-induced increases in APT (10.4%; *d* = 1.360) following 4 days of maximal, reciprocal, concentric, isokinetic forearm EXT and FLEX training at 180° s^−1^ (Figure 1), which reflected a large effect size. Previous studies [9,11,25,26,53] that examined the effects of very short-term resistance training (ranging from 2 training sessions in 1 week to 9 sessions in 2 weeks) have reported mixed findings regarding strength adaptations For example, in untrained men, Beck et al. [24] reported no changes in peak torque at 60°·s^−1^, 180°·s^−1^, and 300°·s^−1^ following 2 days of reciprocal, concentric, isokinetic forearm EXT and FLEX training with 6 sets of 10 repetitions at 180°·s^−1^. Traylor et al. [9,11], however, reported training-induced increases in peak torque at 60°·s^−1^, 180°·s^−1^, and 300°·s^−1^ following 3 days of non-reciprocal, concentric, isokinetic forearm FLEX training with 5 sets of 10 repetitions at 60°·s^−1^ in untrained men, but not in untrained women [11,25]. In the present study, however, 4 days of reciprocal isokinetic training increased APT in untrained women, while 2 or 3 days of training did not. The findings suggested that differences in the number of training days and/or the repetition volume (total number of repetitions performed) can influence the early-phase training-induced adaptations. For example, in the present study, a repetition volume of 180 repetitions following 3 days of training (60 repetitions per day) had no effect on APT, which was consistent with the findings of Traylor et al. [11,25] who reported that no changes in peak torque with a lower repetition volume of 150 repetitions over 3 days of isokinetic training in untrained women. Across 4 days of training in the present study, however, a repetition volume of 240 repetitions was performed, which resulted in an increase in APT in untrained women. Thus, in untrained women, the present findings and those of Traylor et al. [11,25] indicated that 240 repetitions over a 4-day period can increase APT, while 150 to 180 repetitions over 3 days cannot. It appears, however, that the repetition volume required to increase APT may differ between men and women. For example, previous studies [9,11,24,25] have reported increases in peak torque in men with a repetition volume of 150 repetitions, while in the present study, women required 240 repetitions to manifest training-induced increases in forearm EXT and FLEX peak torque. Perhaps there are additional interactions among repetition volume, movement velocity, and the sex of the subject that contribute to the time course of strength adaptations during the early-phase of resistance training. In addition, to examine the contributions of days of training versus repetition volume on early phase adaptations in strength, future studies should compare the training-induced effects of 240 repetitions performed over 2 or 3 days of training versus 4 days of training.

#### 3.2.2. Rate of Velocity Development

Movement velocity influences the torque produced during each repetition, and therefore, may affect training adaptations [54,55,56,57]. For example, Beck et al. [24] reported no changes in isokinetic forearm EXT and FLEX peak torque at 60, 180, and 300°·s^−1^ following 2 days of training at 180°·s^−1^. Traylor et al. [9], however, reported increases in isokinetic forearm FLEX peak torque (13% to 17%) at testing velocities of 60, 180, and 300°·s^−1^ following 3 days of training at 60°·s^−1^. It is possible that the differences in the findings of Beck et al. [24] versus Traylor et al. [9] were due to the different training velocities used in each study. Perhaps, training at 60°·s^−1^ [9] allowed both slow- and fast-twitch fibers to contribute to torque production, while when training at 180°·s^−1^ [24], some slow-twitch fibers could not contract rapidly enough to contribute to torque production [58,59]. Thus, training at 180°·s^−1^ may have limited the training stimulus for some slow-twitch fibers and, therefore, limited the magnitude of the training adaptations. This, however, was not consistent with the results of Prevost et al. [8], who reported no changes in isokinetic leg EXT peak torque for the group that trained at 60°·s^−1^, but reported increases in peak torque for the group that trained at 270°·s^−1^. Furthermore, Brown and Whitehurst [26] attributed the results of Prevost et al. [8] to the failure to control for the acceleration phase of the movement, which can produce artificial increases in torque [27]. Therefore, in the present study, the RVD was assessed to determine the time required to reach the load range phase before quantifiable torque was measured. In the present study, while APT increased for all testing velocities, the training-induced adaptations in RVD decreased (reached the preset velocity faster) with large effect sizes at 180°·s^−1^ (22.0%; *d* = 1.016) and 300°·s^−1^ (15.9%; *d* = 1.153), but not at 60°·s^−1^ following 4 days of training at 180°·s^−1^ for the trained and non-trained arm. These results were consistent with previous studies [26,30,33] that reported training-induced decreases in RVD at 240°·s^−1^ to 300°·s^−1^. Brown et al. [52] and Brown and Whitehurst [26] suggested, however, that some isokinetic dynamometers may not be sensitive enough to detect changes in limb acceleration at slow velocities and, therefore, recommended not using testing velocities of ≤60°·s^−1^ to assess training-induced changes in RVD. Collectively, findings from the present and previous studies [26,30,33] indicated that training at moderate to fast velocities (128°·s^−1^ to 400°·s^−1^) decreased RVD only at testing velocities ≥ 180°·s^−1^ and further suggested that the early-phase adaptations to resistance training were velocity specific.

#### 3.2.3. Average Power

**Figure 2:**
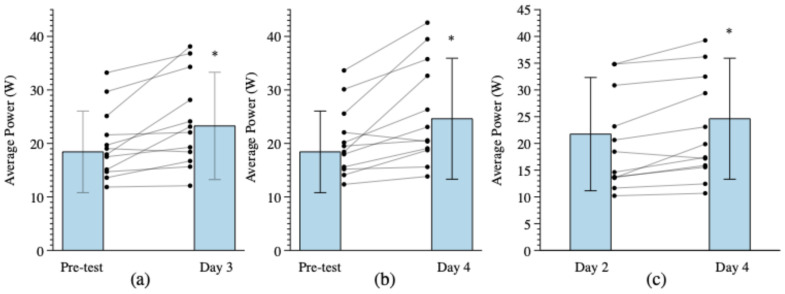
Trained arm marginal means (±SD) and individual spaghetti graphs for average power (collapsed across Velocity and Muscle Action) at pre-test and following Day 3 (**a**) and Day 4 (**b**) of training, as well as at Day 2 and following Day 4 of training (**c**). * Day 3 > pre-test (*p* = 0.037). * Day 4 > pre-test (*p* = 0.023). * Day 4 > Day 2 (*p* = 0.019).

In the present study, there were training-induced increases in AP after 3 (26.6%; *d* = 0.975) and 4 (33.7%; *d* = 1.052) days of training in the trained arm (Figure 2), both of which reflected large effect sizes. There were no changes in AP, however, in the non-trained arm. These findings were not consistent with those of Traylor et al. [11], who reported no changes in AP at 60 or 180°·s^−1^ following 3 days of non-reciprocal, concentric, isokinetic forearm FLEX training at 60°·s^−1^ in untrained women. Perhaps the reciprocal training in the present study, which involved rapid transitions from the antagonist concentric muscle action to the agonist concentric muscle action, increased AP, while the non-reciprocal concentric muscle actions used by Traylor et al. [11] did not. It has been demonstrated that reciprocal muscle actions produce greater torque and muscle excitation than non-reciprocal concentric muscle actions, and the differences in torque production between movement types increase with velocity, possibly due to contributions from the stretch-reflex pathway [7,60]. The rapid lengthening of the agonist muscle during a reciprocal movement can stimulate muscle Ia afferent neurons and, thereby, potentiate the torque production of the agonist muscle during the subsequent repetition [61]. The reciprocal nature of the muscle action, therefore, may influence power production, possibly due to the enhanced torque production from contributions of antagonist inhibition and agonist pre-activation [60,62]. Furthermore, the AP in the present study was calculated as the average integrated area under the torque-time curve and, therefore, depended upon the torque produced throughout the load range of the muscle action and the RVD. Thus, an interaction between training-induced increases in torque production and decreases in RVD, which expanded the load range of the muscle actions, likely contributed to the increases in AP [63].

#### 3.2.4. Potential Mechanisms

Previous studies [3,53,55,64,65,66,67] have attributed the early-phase adaptations to resistance training to neural mechanisms, including improved motor learning, increased muscle excitation, and higher motor unit discharge rates. For example, Akima et al. [53] reported increases in isokinetic leg EXT peak torque over a 2-week period without an increase in muscle cross-sectional area and, thus, attributed the increase in peak torque to increased muscle excitation. Increases in muscle excitation suggest the potential for greater motor unit recruitment, perhaps due to reduced relative recruitment thresholds and/or increased net excitatory input to the motor neuron pool [64]. Previous studies [3,6,24,66,68] that assessed the early-phase neuromuscular mechanisms using electromyography (EMG), however, reported no changes in agonist EMG amplitude, and therefore muscle excitation, following 2 or 3 days of resistance training. Tillin et al. [68], however, reported reduced coactivation based on the relationship for agonist versus antagonist EMG amplitudes following 4 weeks of unilateral leg EXT isometric resistance training, which may have allowed for greater expression of torque from the agonist muscle [68]. It has also been suggested that increases in motor unit discharge rates contribute to training-induced increases in explosive strength [55,64]. Therefore, the early-phase adaptations in APT, RVD, and AP in the present study may be explained by the underlying neural mechanisms that modulate torque and power production of both the agonist and antagonist muscles.

### 3.3. Training Responses in the Non-Trained Arm

It has been suggested that early-phase neural adaptations to exercise-training may be related to the process of learning the motor task [3,8,44,66,69,70]. For example, Kamen and Knight [66] assessed the early-phase of neuromuscular adaptations using high-density surface EMG and reported increases (19%) in motor unit discharge rates between the first and second familiarization sessions (separated by 1 week) of isometric leg EXT. Therefore, it is possible that the increases in APT and AP, and the reductions in RVD in the present study were the result of learning to perform the specific forearm EXT and FLEX motor tasks. Furthermore, in the present study, there were parallel reductions in RVD for the trained and non-trained arm, which suggested a cross-education adaptation. These results were consistent with those of Lee et al. [44], who reported increased acceleration of the first dorsal interosseous muscle in the trained and non-trained hand following a single session of unilateral ballistic training. In addition, Lee et al. [44] reported increases in corticospinal excitability in the contralateral and ipsilateral hemispheres relative to the training hand. It is possible that the 4 training sessions over the 2-week period in the present study may have induced similar neurological increases within the contralateral and ipsilateral hemispheres, and therefore, stimulated a learning effect in the trained and non-trained arm to move faster. It is also possible that the verbal cue given to the subjects during the repeated testing sessions to ‘push and pull as hard and as fast as possible’ [71] led to similar neural adaptations within the contralateral hemisphere of the dominant, non-trained arm. There were, however, no cross-education adaptations for APT and AP in the dominant, non-trained arm following the training. These results may suggest that the mechanisms underlying cross-education adaptations for RVD are different than APT and AP. This hypothesis requires additional research. The cross-education results for APT and AP were consistent with those of Farthing et al. [48], who reported unidirectional cross-education increases in peak torque only when training the dominant arm. In addition, the present findings were not consistent with those of previous studies [68,72,73,74] that reported increased strength and power in the non-trained limb following ≥4 weeks (12 to 24 training sessions) of unilateral resistance training. It is possible, however, that the length of the present study (4 sessions over 2 weeks) may have been too short for training-induced increases in APT and AP to be realized in the dominant, non-trained arm [39]. Future studies should consider examining early phase cross-education adaptations with more training sessions (>4) over a longer period of time (≥2 weeks).

### 3.4. Limitations and Future Directions

The findings in the present study have limitations. For example, the effect size used for the a priori power analysis derived from an 8-week training intervention [30], which may not accurately reflect the magnitude expected over a 4-day training period. Therefore, the present study may have been underpowered for detecting true effects. The MD reported in the present study exceeded the group mean training-induced changes for all variables. These findings should be considered when interpreting the meaningfulness of the significant mean differences. In addition, the results were limited to untrained women. Previous studies [9,11,25] have demonstrated sex-related differences in early-phase adaptations to resistance training, and therefore, the present findings cannot be extended to other populations. Furthermore, the current findings were limited to isokinetic resistance training at 180°·s^−1^ with reciprocal muscle actions, while previous forearm FLEX very short-term isokinetic resistance training studies [11,25] trained at 60°·s^−1^ with non-reciprocal, concentric muscle actions. Future studies should compare the early-phase adaptations following 2, 3, and 4 days of isokinetic training at multiple velocities with reciprocal and non-reciprocal concentric muscle actions in untrained men. In addition, the training data (e.g., total work performed) in the present study were not included in the analysis; therefore, the training quality across the four training sessions cannot be determined. Future studies should consider including training data to improve the interpretation of the results. Future research should also consider the distribution of repetition volume over 2, 3, and 4 days of training to further compare the contributions of training frequency and training volume to the early-phase adaptations to resistance training, and should examine the time course of early-phase cross-education adaptations from training the dominant versus non-dominant arm. In addition, the present study alludes to various neuromuscular responses; however, these conclusions are only hypothetical. Future studies should consider including neuromuscular assessments (such as EMG) to further elucidate the neural adaptations that result from very short-term resistance training in the trained arm as well as cross-education effects in the non-trained arm.

## 4. Materials and Methods

### 4.1. Experimental Design

**Figure 3:**
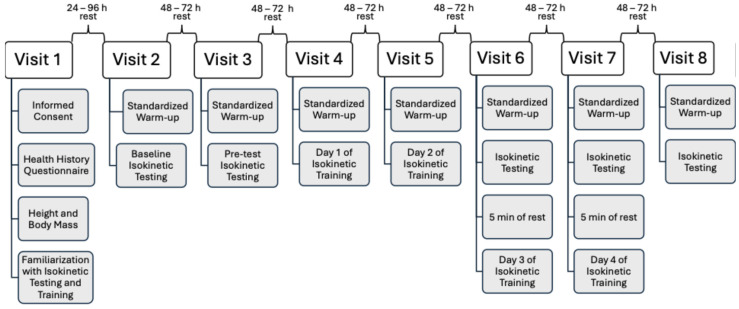
Time course of procedures for the isokinetic testing and training visits.

The present study investigated the effects of 2, 3, and 4 days of unilateral, reciprocal, concentric, isokinetic forearm EXT and FLEX training at 180°·s^−1^ on APT, AP, and RVD in untrained women. The subjects visited the laboratory on 8 separate occasions with 24 to 96 h of rest between visits (Figure 3). During the first visit, the subjects were informed of the benefits and risks of participation in the study, signed a written Informed Consent form, completed a Health History Questionnaire, and were familiarized with the isokinetic testing procedures. During visits 2 and 3, the subjects performed a standardized warm-up followed by the isokinetic testing procedures. Visits 2 and 3 were used for the reliability of the isokinetic testing and to establish pre-testing values. During visits 4 (Day 1 of training) and 5 (Day 2 of training), the subjects completed the same standardized warm-up followed by the isokinetic training protocol with no testing. During visits 6 (Day 3 of training) and 7 (Day 4 of training), the subjects completed the standardized warm-up, isokinetic testing procedures, 5 min of rest, and then performed the isokinetic training protocol. During visit 8 (following Day 4 of training), the subjects returned to the laboratory to perform the same isokinetic testing procedures as visit 2 (baseline), visit 3 (pre-test), visit 6 (following Day 2 of training), and visit 7 (following Day 3 of training). The testing procedures were performed with both the dominant and non-dominant arm (based on throwing preference), while training was performed with only the non-dominant arm.

### 4.2. Subjects

An a priori power analysis (G*Power 3.1, Heinrich-Heine-Universität Düsseldorf) indicated that a minimum of *n* = 6 subjects was required to detect an effect of *f* = 0.59 with *a* = 0.05 and power = 0.80. The effect size was estimated from a previous study [30], which reported a mean difference of *d* = 0.65 between pre- and post-training for RVD in the high-velocity group. The effect size was converted to Cohen’s *f* for use in the repeated-measures ANOVA power analysis. In the present study, 12 women (mean ± SD: age: 21.7 ± 1.2 yrs; height: 167.9 ± 5.2 cm; body mass: 64.7 ± 6.8 kg) volunteered to participate. The subjects were untrained in upper body resistance exercise (<1 d/wk^−1^ for the last 3 months) and reported no upper body pathologies or injuries that would affect their forearm FLEX and EXT performance. The subjects in the present study were part of a larger, multiple-independent- and dependent-variable investigation; however, none of the current data have been previously published. The ethical principles in the present study were approved by the University of Nebraska-Lincoln’s Institutional Review Board for Human Subjects (IRB Approval #: 20220421791FB). All the subjects were informed about the experimental procedures, potential risks of participating, and provided their written Informed Consent prior to participating in any testing.

### 4.3. Procedures

#### 4.3.1. Isokinetic Testing

Isokinetic testing occurred during visits 2 (baseline), 3 (pre-test), 6 (following Day 2 of training), 7 (following Day 3 of training), and 8 (following Day 4 of training) to determine maximal, unilateral, reciprocal, concentric, isokinetic forearm EXT and FLEX peak torque, power, and RVD over three repetitions at 60°·s^−1^, 180°·s^−1^, and 300°·s^−1^ (Figure 3). All testing procedures were performed with the dominant and non-dominant arm. The subjects were positioned on a Biodex System 4 Pro (Biodex Medical Systems, Inc., Shirley, NY, USA) according to the manufacturer’s guidelines. Prior to testing, the subjects performed a standardized warm-up that consisted of 2 sets of 3 s submaximal (50 and 70% of perceived maximal effort) isometric contractions for forearm EXT and FLEX at an elbow joint angle of 90° and then completed 1 set of 5, submaximal (self-selected), reciprocal, concentric, isokinetic forearm EXT and forearm FLEX repetitions at 60°·s^−1^, 180°·s^−1^, and 300°·s^−1^. Following the standardized warm-up, the subjects performed 3 maximal, reciprocal, concentric, isokinetic EXT and FLEX repetitions at each velocity (60°·s^−1^, 180°·s^−1^, and 300°·s^−1^) through a joint range of motion of ~135° (full EXT = ~180°; full FLEX = ~45°) and were instructed to ‘push and pull as hard and as fast as possible’ for all forearm EXT and FLEX repetitions, respectively. The subjects were given 2 min of rest between the warm-up and the testing protocol. The isokinetic testing velocities were performed in a randomized order with 10 s rest between testing velocities. The subjects received strong verbal encouragement during the isokinetic testing to ensure maximal effort was given for all 3 repetitions. The AP value was provided by the Biodex System 4 Pro as the averaged integral of the torque-time curve across the 3 repetitions. To be consistent with the selection of the AP values, the APT values were also obtained from the Biodex System 4 Pro as the averaged value across the 3 repetitions. The RVD was measured as the time required to reach the load range phase before quantifiable torque can be measured. The fastest RVD from the 3 repetitions was provided by the Biodex System 4 Pro and used for subsequent analyses.

#### 4.3.2. Isokinetic Training

Isokinetic training sessions occurred during visits 4 through 7. Prior to engaging in the isokinetic training protocol, the subjects completed the same standardized warm-up that preceded testing. Following the standardized warm-up and 2 min rest period, the subjects performed 6 sets of 10 maximal, unilateral, reciprocal, concentric, isokinetic forearm EXT and FLEX at 180°·s^−1^ with the non-dominant arm only. Two minutes of rest were given between sets. The subjects were instructed to ‘push and pull as hard and as fast as possible’ and received strong verbal encouragement during each set of the training protocol to ensure maximal effort was given during all 10 repetitions.

### 4.4. Statistical Analysis

The test–retest reliability for the baseline (visit 2) versus pre-test (visit 3) for APT, AP, and RVD at 60°·s^−1^, 180°·s^−1^, and 300°·s^−1^ for visits 2 and 3 were assessed with repeated measures ANOVAs to evaluate the systematic error and a mean-rating (*k* = 2), absolute agreement, 2-way mixed effects model was used to determine the ICC [51]. The magnitudes of the ICC values were interpreted using the following classifications [75]: poor (<0.40); fair (0.40–0.59); good (0.60–0.74); and excellent (0.75–1.00). The SEM was calculated as $SEM=SD\sqrt{1-ICC}$ [51]. The MD was calculated as $MD=SEM\times 1.96\times \sqrt{2}$ [51]. The mean differences for pre-test versus following Day 2, Day 3, and Day 4 of training for APT, AP, and RVD were determined using separate 2 (Arm: training arm and non-training arm) × 2 (Muscle Action: forearm EXT and FLEX) × 3 (Velocity: 60°·s^−1^, 180°·s^−1^, and 300°·s^−1^) × 4 (Time: pre-test, Day 2, Day 3, and Day 4) repeated measures ANOVAs. Significant interactions and main effects were analyzed using post hoc pairwise comparisons with Bonferroni correction to control for inflated Type I error rates. The effect sizes for the interaction and main effects from the repeated measures ANOVAs were calculated as partial eta squared ($\eta_{p}^{2}$) and interpreted as 0.01 = small, 0.06 = medium, and 0.14 = large [76]. The effect sizes for the post hoc pairwise comparisons were calculated as Cohen’s *d* and were interpreted as 0.2 = small, 0.5 = medium, and 0.8 = large [76]. Statistical significance was set at *α* = 0.05. All statistical analyses were completed using IBM SPSS version 29 (SPSS Inc., Armonk, NY, USA). Figures were developed using DataGraph version 5.5 (Visual Data Tools Inc., Chapel Hill, NC, USA).

## 5. Conclusions

In summary, the results of the present study indicated that untrained women required a minimum of 4 days of unilateral, reciprocal, concentric, isokinetic forearm EXT and FLEX resistance training to manifest increases in APT and decreases in RVD in the trained arm, while increases in AP in the trained arm required only 3 days. In the present study, the large repetition volume (240 total repetitions) as well as the nature of the reciprocal muscle actions may have contributed to the early-phase increases in APT and AP. The rapid lengthening of the agonist muscle during reciprocal actions may potentiate the torque production for the subsequent repetition and, therefore, enhance power production. There were, however, no cross-training adaptations for APT or AP in the non-trained arm, possibly due to the length of training and/or training the non-dominant arm. In addition, the RVD at 180°·s^−1^ and 300°·s^−1^ decreased following 4 days of training for the trained and non-trained arm. The verbal cueing given to the subjects during the testing visits may have facilitated a learning outcome for the non-trained arm to move faster. The RVD at 60°·s^−1^, however, did not change following 2, 3, or 4 days of training for the trained and non-trained arm. It has been suggested [26,52] that some isokinetic dynamometers may not be sensitive enough to detect training-induced changes in RVD at slow velocities, and therefore, testing velocities ≤ 60°·s^−1^ are not recommended.

## Figures and Tables

**Table 1:** Reliability of forearm extension data for the 3-repetition maximal isokinetic reciprocal forearm extension and flexion test at each preset velocity for the trained arm and non-trained arm.

Trained Arm	Variable	Visit 1	Visit 2	*p*	ICC	ICC_95%_	SEM	MD
60° s^−1^	APT (Nm)	17.8 ± 4.5	19.8 ± 6.3	0.064	0.889	0.584–0.969	2.7	7.6
	AP (W)	13.0 ± 3.7	14.4 ± 4.5	0.038	0.920	0.652–0.978	1.8	4.9
	RVD (ms)	95.0 ± 20.2	87.5 ± 10.9	0.145	0.661	−0.049–0.899	14.3	39.7
180° s^−1^	APT (Nm)	13.4 ± 4.0	14.6 ± 3.9	0.042	0.928	0.690–0.980	1.6	4.4
	AP (W)	19.6 ± 9.0	22.4 ± 8.7	0.060	0.919	0.684–0.977	3.8	10.5
	RVD (ms)	195.0 ± 38.6	185.8 ± 26.3	0.312	0.765	0.224–0.932	23.9	66.2
300° s^−1^	APT (Nm)	8.6 ± 3.8	9.3 ± 3.5	0.125	0.954	0.837–0.987	1.2	3.2
	AP (W)	10.4 ± 8.3	11.8 ± 7.8	0.089	0.970	0.887–0.992	2.1	5.7
	RVD (ms)	300.0 ± 42.2	295.8 ± 51.6	0.674	0.877	0.571–0.965	24.4	67.7
**Non-Trained Arm**								
60° s^−1^	APT (Nm)	22.0 ± 6.4	22.0 ± 5.9	0.988	0.907	0.669–0.973	2.8	7.7
	AP (W)	17.8 ± 5.7	17.9 ± 5.8	0.896	0.916	0.705–0.976	2.0	5.5
	RVD (ms)	77.5 ± 47.6	77.5 ± 12.3	1.000	0.429	−1.274–0.842	38.8	107.6
180° s^−1^	APT (Nm)	17.5 ± 5.6	17.9 ± 5.3	0.612	0.952	0.837–0.986	1.8	4.9
	AP (W)	29.2 ± 11.5	31.2 ± 11.9	0.057	0.975	0.895–0.993	2.7	7.6
	RVD (ms)	159.2 ± 53.5	140.8 ± 48.7	0.378	0.283	−1.503–0.794	65.0	180.1
300° s^−1^	APT (Nm)	12.8 ± 4.9	13.4 ± 4.4	0.432	0.944	0.813–0.984	1.6	4.5
	AP (W)	21.7 ± 13.8	23.9 ± 11.4	0.147	0.959	0.858–0.988	3.8	10.5
	RVD (ms)	225.8 ± 47.9	225.0 ± 26.3	0.935	0.791	0.238–0.941	20.3	56.3

Values are reported as mean ± SD. *p* = probability value from the ANOVA for systematic error; ICC = intraclass correlation coefficient; ICC_95%_ = ICC 95% confidence interval; SEM = standard error of the measurement; MD = minimal difference; APT = average peak torque; AP = average power; RVD = rate of velocity development.

**Table 2:** Reliability of forearm flexion data for the 3-repetition maximal isokinetic reciprocal forearm extension and flexion test at each preset velocity for the trained arm and non-trained arm.

Trained Arm	Variable	Visit 1	Visit 2	*p*	ICC	ICC_95%_	SEM	MD
60° s^−1^	APT (Nm)	27.1 ± 6.1	27.2 ± 6.0	0.846	0.985	0.947–0.996	1.1	3.0
	AP (W)	20.3 ± 5.2	20.0 ± 4.4	0.624	0.943	0.805–0.984	1.7	4.7
	RVD (ms)	161.7 ± 60.5	133.3 ± 45.3	0.39	0.788	0.242–0.939	33.4	92.6
180° s^−1^	APT (Nm)	21.6 ± 5.5	22.3 ± 5.5	0.015	0.992	0.948–0.998	0.7	2.0
	AP (W)	26.0 ± 13.3	28.4 ± 12.4	0.033	0.978	0.889–0.994	2.8	7.8
	RVD (ms)	331.7 ± 147.1	308.3 ± 95.9	0.404	0.855	0.512–0.958	70.2	194.5
300° s^−1^	APT (Nm)	15.3 ± 6.2	15.7 ± 6.2	0.634	0.951	0.831–0.986	2.0	5.6
	AP (W)	12.8 ± 10.6	13.6 ± 9.7	0.429	0.972	0.906–0.992	2.5	7.0
	RVD (ms)	496.7 ± 138.8	492.5 ± 191.8	0.901	0.890	0.610–0.969	82.0	227.3
**Non-Trained Arm**								
60° s^−1^	APT (Nm)	29.1 ± 5.3	30.3 ± 6.5	0.180	0.939	0.793–0.982	2.2	6.0
	AP (W)	21.9 ± 5.3	23.8 ± 5.8	0.066	0.897	0.615–0.971	2.7	7.4
	RVD (ms)	110.0 ± 22.7	95.0 ± 21.0	0.043	0.601	−0.157–0.878	21.6	59.8
180° s^−1^	APT (Nm)	24.0 ± 4.9	24.3 ± 6.1	0.703	0.930	0.758–0.980	2.2	6.0
	AP (W)	32.5 ± 12.9	37.3 ± 14.7	0.011	0.939	0.609–0.985	5.1	14.2
	RVD (ms)	243.3 ± 84.4	239.2 ± 91.1	0.878	0.686	−0.169–0.911	72.7	201.5
300° s^−1^	APT (Nm)	19.4 ± 4.5	19.4 ± 4.7	0.982	0.935	0.771–0.981	1.7	4.8
	AP (W)	20.3 ± 14.6	21.4 ± 13.2	0.539	0.955	0.848–0.987	4.4	12.1
	RVD (ms)	401.7 ± 126.9	399.2 ± 94.3	0.917	0.873	0.548–0.964	75.5	209.3

Values are reported as mean ± SD. *p* = probability value from the ANOVA for systematic error; ICC = intraclass correlation coefficient; ICC_95%_ = ICC 95% confidence interval; SEM = standard error of the measurement; MD = minimal difference; APT = average peak torque; AP = average power; and RVD = rate of velocity development.

## Data Availability

The datasets presented in the article are not readily available but would be made available upon reasonable request to the authors.

## Abbreviations

The following abbreviations were used in this manuscript:

APT	Average Peak Torque
RVD	Rate of Velocity Development
AP	Average Power
FLEX	Flexion
EXT	Extension
DCER	Dynamic Constant External Resistance
ICC	Interclass Correlation Coefficient
ICC_95%_	95% Confidence Interval for the Interclass Correlation Coefficient
SEM	Standard Error of Measurement
MD	Minimal Difference
EMG	Electromyography
